# SARS-CoV-2 Serum Neutralization Assay: A Traditional Tool for a Brand-New Virus

**DOI:** 10.3390/v13040655

**Published:** 2021-04-10

**Authors:** Giulia Matusali, Francesca Colavita, Daniele Lapa, Silvia Meschi, Licia Bordi, Pierluca Piselli, Roberta Gagliardini, Angela Corpolongo, Emanuele Nicastri, Andrea Antinori, Giuseppe Ippolito, Maria Rosaria Capobianchi, Concetta Castilletti, INMI COVID-19 Laboratory Team

**Affiliations:** National Institute for Infectious Diseases “L. Spallanzani” IRCCS, Via Portuense 292, 00149 Rome, Italy; giulia.matusali@inmi.it (G.M.); francesca.colavita@inmi.it (F.C.); silvia.meschi@inmi.it (S.M.); licia.bordi@inmi.it (L.B.); pierluca.piselli@inmi.it (P.P.); roberta.gagliardini@inmi.it (R.G.); angela.corpolongo@inmi.it (A.C.); emanuele.nicastri@inmi.it (E.N.); andrea.antinori@inmi.it (A.A.); giuseppe.ippolito@inmi.it (G.I.); maria.capobianchi@inmi.it (M.R.C.); concetta.castilletti@inmi.it (C.C.)

**Keywords:** SARS-CoV-2, neutralizing antibodies, protective immunity, serology

## Abstract

SARS-CoV-2 serum neutralization assay represents the gold standard for assessing antibody-mediated protection in naturally infected and vaccinated individuals. In the present study, 662 serum samples collected from February 2020 to January 2021 from acute and convalescent COVID-19 patients were tested to determine neutralizing antibody (NAb) titers using a microneutralization test (MNT) for live SARS-CoV-2. Moreover, anti-SARS-CoV-2 IgG, IgA, and IgM directed against different viral antigens were measured by high-throughput automated platforms. We observed higher levels of NAbs in elderly (>60 years old) individuals and in patients presenting acute respiratory distress syndrome. SARS-CoV-2 NAbs develop as soon as five days from symptom onset and, despite a decline after the second month, persist for over 11 months, showing variable dynamics. Through correlation and receiver operating characteristic (ROC) curve analysis, we set up a testing algorithm, suitable for the laboratory workload, by establishing an optimal cutoff value of anti-SARS-CoV-2 IgG for convalescent plasma donors to exclude from MNT samples foreseen to have low/negative NAb titers and ineligible for plasma donation. Overall, MNT, although cumbersome and not suitable for routine testing of large sample sizes, remains the reference tool for the assessment of antibody-mediated immunity after SARS-CoV-2 infection. Smart testing algorithms may optimize the laboratory workflow to monitor antibody-mediated protection in COVID-19 patients, plasma donors, and vaccinated individuals.

## 1. Introduction

When the novel coronavirus, first identified in December 2019 in Wuhan, China, was recognized as the etiologic agent of a severe acute respiratory syndrome (later named SARS-CoV-2), molecular assays were the first tool developed and commercialized for the diagnostic response to the outbreak. These assays measure acute infection, detecting viral RNA in respiratory samples, and are essential for individuals who require healthcare by tracing the infection in the population and reducing viral transmission. In contrast to molecular tests, serology should not be used to diagnose acute SARS-CoV-2 infection, and only on rare occasions might it be useful as an adjunct diagnostic test [1].

The need for SARS-CoV-2 serological testing became more urgent soon after the very early stage of the SARS-CoV-2 pandemic. Testing should function as an instrument to: (i) measure the extent of infection in the population; (ii) understand antibody-mediated response kinetics and duration; (iii) investigate antibody-mediated immune protection.

For SARS-CoV-2 seroprevalence and antibody kinetics studies, high-throughput platforms (i.e., enzyme-linked immunosorbent assay (ELISA) and chemiluminescent immunoassay (CLIA)) able to detect IgG, as well as IgA and IgM, have been widely used, allowing for faster turnaround times and easy expansion of testing capacity.

However, these tests, although able to detect the ability of antibodies to bind viral proteins/structures, are not informative on their functional properties, i.e., the ability to neutralize the infectivity of viral particles. To this end, a biological assay is needed, namely a serum neutralization test, and several options for the test format, including microneutralization test (MNT), have been developed.

In the traditional neutralization assay, a defined quantity of virus (usually 100 TCID50) is mixed with serial dilutions of the test serum. Following incubation, to allow the potential neutralization activity, virus/serum mixtures are inoculated into susceptible culture cells. The cells are incubated at a temperature suitable for viral growth for 48 to 72 h and are then examined for the production of a virus-induced cytopathic effect (CPE) (or some other indicator of viral growth). The performance of traditional MNT requires specialized personnel, a laboratory turnaround of more than 72 h, and, as for SARS-CoV-2, high biocontainment laboratories (i.e., BSL-3). The use of pseudo-typed viruses may avoid the requirement for high-level biosafety laboratories; nevertheless, virus serum neutralization test remains the gold standard for determining antibody protective efficacy.

In the case of SARS-CoV-2, a key target for neutralizing antibodies (NAbs) lies in the receptor-binding domain (RBD) of the Spike (S) viral protein [2]. Based on this evidence, some tests have been developed to measure anti-RBD IgG. These assays have demonstrated a good correlation with NAb titers; however, none of these tests can currently replace MNT for the functional evaluation of antibodies [3].

Neutralization assay has four main applications in the management of the SARS-CoV-2 outbreak. Using this tool, we can assess the presence and duration of antibody-mediated protection in naturally infected individuals, screen convalescent plasma (CP) preparations for donation, test the efficacy of immunotherapy (i.e., CP or monoclonal antibodies), and analyze NAb titers following vaccination.

Here, we present the results of MNT performed on serum samples collected from February 2020 to January 2021 at the Laboratory of Virology of the National Institute for Infectious Diseases “L. Spallanzani” (INMI) in Rome, Italy, to assess protective antibody dynamics in naturally infected individuals and to screen convalescent plasma for immunotherapeutic intervention. Samples were also analyzed with commercial assays detecting antibodies against different viral antigens, and the correlation with MNT was evaluated.

The presence and duration of protective immunity due to natural infection or therapeutic/prophylactic intervention (e.g., immunotherapy or vaccination) and the availability of adequate serological assays to predict protection will affect future (re)infection, viral transmission, COVID-19 immunization campaign, and clinical management.

## 2. Material and Methods

### 2.1. Study Group

In total, 763 serum samples from 662 symptomatic COVID-19 patients were analyzed in this study. The samples were collected from adult SARS-CoV-2-infected acute and convalescent patients, the latter group either recruited for clinical follow-up or screened for potential CP donation.

The median age was 49 years old (Interquartile-range IQR 38–57) and 73% of individuals were male (gender was not reported for two patients).

The date of symptom onset was known for 390 individuals, and the time of collection varied from 2 to 323 days from symptom onset (fso). NAbs were measured in 66 (from 54 patients) samples collected >6 months fso and 35 samples were collected 8–11 months fso. Longitudinal sampling of serum samples >6 months fso was performed for 45 patients to investigate NAb kinetics, appearance, and persistence.

As a marker of disease severity, we used the lower P/F ratio (arterial pO2 divided by the fraction of inspired oxygen (FiO2) expressed as a decimal—the patient is receiving) measured during acute infection. P/F ratio <300 mmHg is diagnostic of mild acute respiratory distress syndrome (ARDS), <200 mmHg is consistent with moderate ARDS, and <100 mmHg indicates severe ARDS [4].

### 2.2. SARS-CoV-2 Microneutralization Assay

Serum samples were heat-inactivated at 56 °C for 30 min and titrated in duplicate in 7 twofold serial dilutions (starting dilution 1:10). Each serum dilution (50 μL) and medium (50 μL) containing 100 TCID50 SARS-CoV-2 (SARS-CoV-2/Human/ITA/PAVIA10734/2020 [5], isolated in March and provided by Fausto Baldanti, Pavia, Italy) were mixed and incubated at 37 °C for 30 min. Subsequently, 96-well tissue culture plates with sub-confluent Vero E6 cell (ATCC, Manassas, Virginia, United States) monolayers were infected with 100 μL/well of virus/serum mixtures and incubated at 37 °C and 5% CO_2_. To standardize inter-assay procedures, positive control samples showing high (1:160) and low (1:40) neutralizing activity were included in each assay session. After 48 h, microplates were observed by light microscope for the presence of CPE. The supernatant of each plate was carefully discarded, and 120 μL of a crystal violet solution (Diapath S.P.A., Martinengo, Italy) containing 2% formaldehyde (Sigma-Aldrich, St. Louis, MI, USA) was added to each well. After 30 min, the fixing solution was removed by washing with tap water, and cell viability was measured by a photometer at 595 nm (Synergy™ HTX Multi-Mode Microplate Reader, Biotek, Winooski, VT, USA). The highest serum dilution inhibiting at least 90% of the CPE was indicated as the neutralization titer. Serum from the National Institute for Biological Standards and Control, Blanche Lane, Ridge, Herts, UK (NIBSC) with known neutralization titer (Research reagent for anti-SARS-CoV-2 Ab NIBSC code 20/130) was used as a reference in MNT.

### 2.3. SARS-CoV-2 Antibody Immunoassays

Three commercial antibody assays were used, according to manufacturer’s protocols: (1) the Abbott SARS-CoV-2 assay on Abbott ARCHITECT^®^ i2000sr, a chemiluminescence microparticle assay (CMIA) detecting anti-Nucleoprotein (anti-N) IgG (index values, i.e., Sample/Cutoff, ≥ 1.4 are considered positive; Abbott Diagnostics, Chicago, IL, USA); (2) the DiaSorin LIAISON^®^ SARS-CoV-2 S1/S2 IgG test on LIAISON^®^ XL analyzers, a chemiluminescence immunoassay (CLIA) detecting anti-S1/S2 IgG (IgG antibody concentrations expressed as arbitrary units, AU/mL ≥ 15 are considered positive, DiaSorin, Saluggia, Italy); (3) an enzyme-linked immunosorbent assay (ELISA) able to detect anti-SARS-CoV-2 IgG, IgM, and IgA (index values S/CO ≥ 1.1 are considered positive; DIESSE, Diagnostica Senese; Siena, Italy). The laboratory turnaround time was 30 min for the CLIA and CMIA, while it was 7 h for ELISA.

### 2.4. Statistical Analysis

Differences in NAb titers were analyzed using Mann–Whitney non-parametric tests; the correlation between antibody levels measured by different assays was evaluated using Spearman analysis (GraphPad Software, La Jolla, CA, USA), and *p*-value < 0.05 was considered statistically significant. Receiver operating characteristic (ROC) analysis was used to establish the optimal cutoff values for anti-SARS-CoV-2 IgG to identify samples with adequate NAb titers, using MedCalc Statistical Software version 19.2.6 (MedCalc Software bv, Ostend, Belgium; https://www.medcalc.org; accessed on 9 April 2021).

## 3. Results

### 3.1. Neutralizing Antibodies in Symptomatic COVID-19 Patients Are Detected Early upon Infection and May Persist Over 11 Months

Overall, 763 serum samples from 662 symptomatic COVID-19 patients were analyzed to explore the correlation of neutralizing antibody response with personal (age and gender) and clinical (disease severity) characteristics of the patients, as well as the duration of neutralizing antibody response.

**Figure 1:**
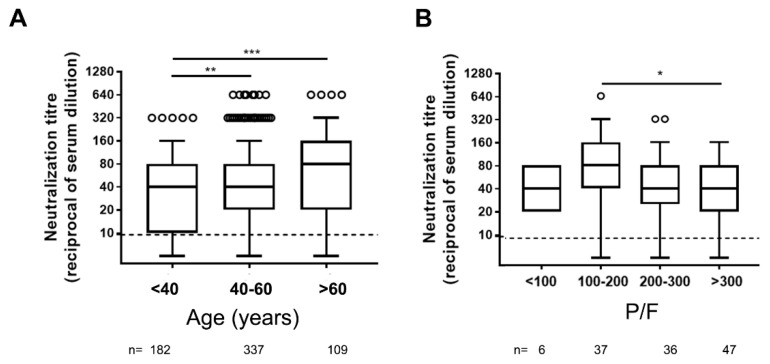
Neutralizing antibody titers in different ages (**A**) and COVID-19 severity (**B**) groups of symptomatic COVID-19 patients. P/F = arterial pO2 divided by the fraction of inspired oxygen (FiO2) used as correlate of diseases severity. Dot line indicates the limit of neutralizing antibody (NAb) quantification, n = number of patients tested. Statistical analysis was performed by Mann–Whitney test *p* values < 0.05, < 0.01, < 0.001 are indicated as *, **, and *** respectively.

Concerning age, significantly higher levels of NAbs were detected in serum samples from elderly individuals (over 60 years old) compared to younger (18–40 and 40–60 years old) adults (Figure 1A).

When we analyzed the potential influence of sex on neutralizing response, we observed lower levels of NAb titers in female individuals; however, this difference was not observed when the comparison was performed on the subgroup of age-matched patients who were hospitalized at INMI (not shown).

The disease severity is another factor potentially associated with higher NAb levels [6,7,8,9,10,11,12]. As a marker of disease severity, we used the P/F ratio (see methods). A weak negative correlation was observed among neutralization titer and P/F ratio (r = −0.19; *p* = 0.029), and significantly higher levels of NAbs were measured in patients with moderate ARDS compared to those not presenting ARDS (P/F > 300) (Figure 1B).

To date, one key question remains unanswered (or only partially investigated) regarding antibody-mediated response: how long does the NAb response last?

To answer this question, we analyzed the presence of NAbs in 475 serum samples for which the time fso was known. This collection covered a wide range of time points, from few days to 11 months fso.

**Figure 2:**
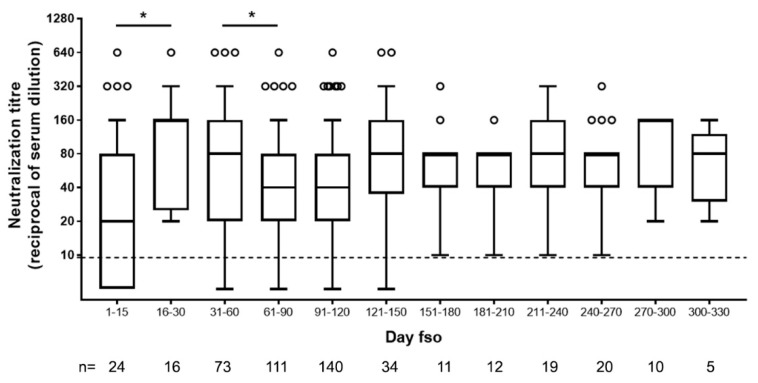
Neutralizing antibody titers in serum samples of SARS-CoV-2-infected patients collected at different time from symptom onset. Dot line indicates limit of NAb quantification. fso = from symptom onset, n = number of patients tested. Statistical analysis was performed by Mann–Whitney test; *p* values < 0.05 is indicated as *.

In accordance with what was previously reported about [13] and expected for an acute viral disease, we observed an initial peak of neutralizing antibody response (day 15–30, up to 1:640) and a slight decline after the second month fso (Figure 2).

Afterward, and until over 270 to 330 days fso (9 and 11 months, respectively), NAb levels remained stable in our cohort (Figure 2).

A neutralizing response was detected as soon as five days fso, and very high levels could be reached in the first weeks of symptoms.

Neutralization titers in serum samples collected less than one month fso showed direct correlation with anti-SARS-CoV-2 total IgG (r = 0.60; *p* = 0.002; n = 24), anti-Spike (anti-S) IgG (r = 0.84; *p* < 0.0001; n = 24), anti-Nucleoprotein IgG (r = 0.74; *p* < 0.0001; n = 25), and anti-SARS-CoV-2 IgA (r = 0.52; *p* = 0.009; n = 24), but not with specific IgM (r = 0.32; *p* = 0.121; n = 24), suggesting a role of both IgG and IgA in the neutralization activity of serum samples (Figure S1).

**Figure 3:**
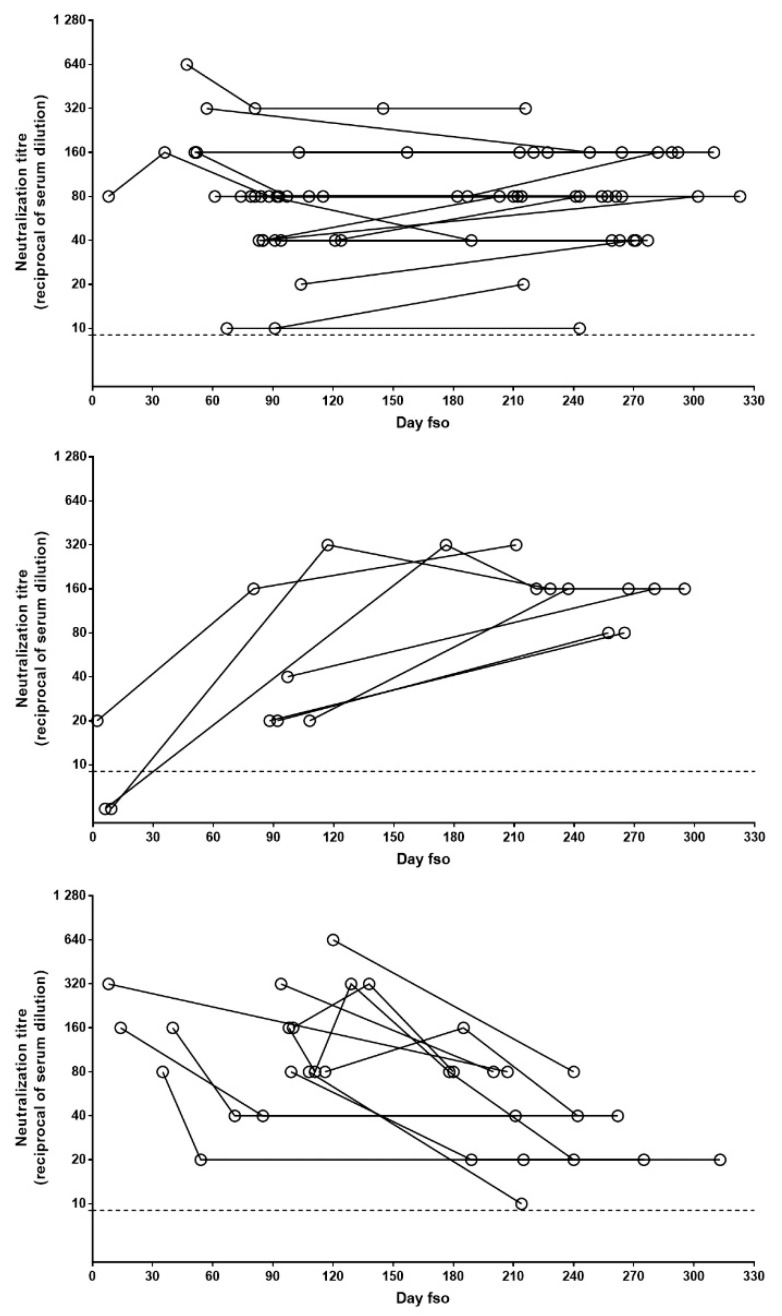
Patterns of NAb persistence. NAb titers in longitudinally collected samples from patients with at least 6 months follow-up post-infection are shown in the panels. Data are grouped according to evolution of NAb levels over time: stable (upper panel), increasing (central panel), or decreasing (lower panel). Each dot represents one serum sample and each line indicates one patient. Dot line indicates limit of NAb quantification. fso = from symptom onset.

To describe the pattern of NAb persistence, we performed further analysis, only including patients with longitudinal sample collection covering at least 6 months (n = 45). Three main patterns, based on the difference between the first and last samples, were observed: stable (i.e., oscillations not exceeding 1 twofold dilution), increasing, and decreasing. In the majority of cases (n = 27; 60%), a stable NAb pattern was observed, while the increasing and decreasing patterns were observed in 7 (15%) and 11 (24%) patients, respectively (Figure 3). In all the patients followed longitudinally, NAb titers never declined to undetectable levels.

### 3.2. Orienting the Choice of the Serological Platform to Assess Ab-Mediated Protection

Another unanswered question regarding antibody-mediated response is: which are the best serological correlates of protection? In other words, neutralization assays are cumbersome and require a long laboratory turnaround time. We need to identify high-throughput quantitative serological tests able to provide a surrogate marker of antibody-mediated protection to be used as a screening step to reduce the workload associated with NAb titration (e.g., for the selection of plasma donors as well as to monitor passive immunization and vaccination).

**Figure 4:**
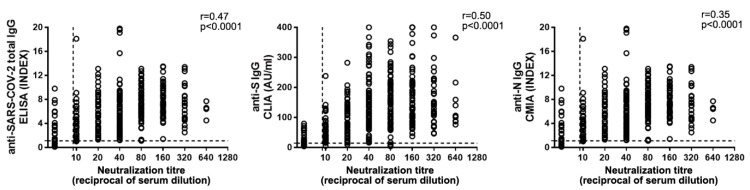
Anti-SARS-CoV2 IgG correlation to NAb titers. Total anti-SARS-CoV-2 (**left** panel), anti-Spike (anti-S) (**central** panel), and anti-Nucleoprotein (anti-N) (**right** panel) IgG levels were compared to NAb titers. Horizontal dot line indicates limit of NAb level quantification, vertical dot line indicates the limit of IgG detection. Spearman’s test was used for correlation analysis.

In order to find the best correlate of antibody (Ab)-mediated protection, we compared NAb levels with anti-SARS-CoV-2 IgG, measured through different platforms (Figure 4). The automated systems available at the INMI laboratory measure anti-SARS-CoV-2 total IgG, anti-N IgG, and anti-S IgG. When we performed correlation analysis of neutralization titers with IgG levels, the highest coefficient was obtained with anti-S IgG (n = 428; r = 0.50; *p* < 0.0001,), while weaker correlations were observed with total (n = 366; r = 0.47; *p* < 0.0001) or anti-N (n = 389; r = 0.36; *p* < 0.0001) IgG (Figure 4).

**Figure 5:**
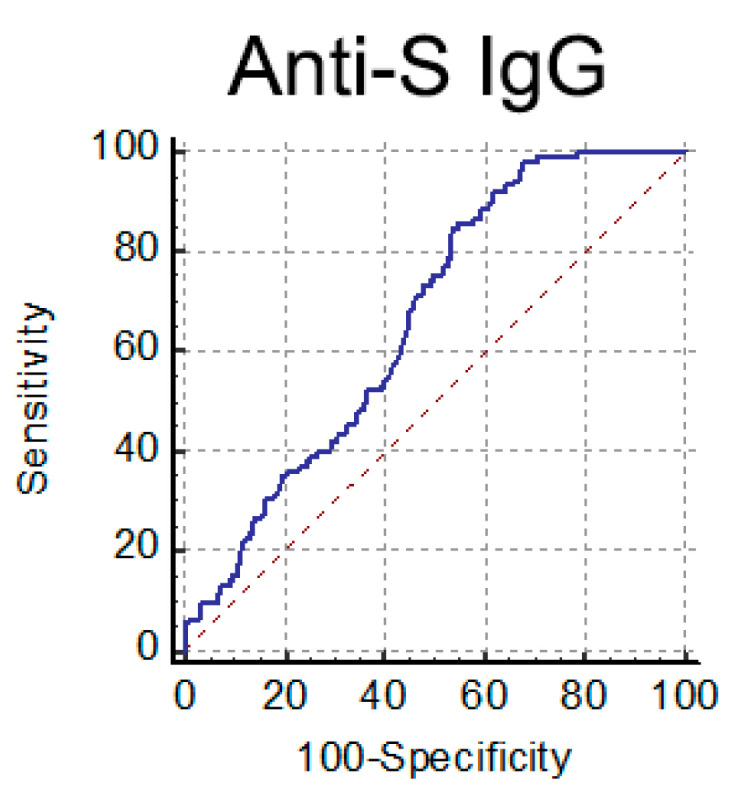
ROC curve analysis performed for anti-S IgG to detect high NAb titer (≥1:160) serum samples.

Based on these data, we chose to use the anti-S IgG test as a pre-screening assay of serum from CP donors. According to Italian guidelines [14], a neutralization titer ≥ 1:160 is a selection criterion for convalescent plasma for treating COVID-19 patients. We tried to establish an IgG threshold value meeting this selection criterion (NAb titer ≥ 1:160). To this end, we analyzed NAb and anti-S IgG titers in 402 serum samples from COVID-19 convalescent individuals (>30 days fso). When we performed a ROC curve analysis (Figure 5), the optimal criterion was obtained with a cutoff of 82.6 AU/mL for anti-S IgG (Sensitivity 84.8%, 95%CI 76.4–91.0; Specificity 46.5%, 95%CI 40.7–52.3).

However, with this cutoff, 14% of potential donors would have been lost (Table 1). For this reason, we decided to adopt an IgG cutoff of 60 AU/mL (sensitivity 99%, 95%CI 94.8–100.0; specificity 29%, 95%CI 24.2–34.8), i.e., a more conservative value, to maximize the identification of adequate plasma donations, decreasing specificity in favor of sensitivity. Indeed, using this cutoff, only 1 out of 105 potential donors (0.95%) would be excluded, while diminishing the workload by 87 NAb tests (Table 1).

## 4. Discussion

The worldwide spread of SARS-CoV-2 has deeply and rapidly affected the health care system, determining the adoption of a number of new and traditional procedures to manage this pandemic situation [15,16].

When a new virus emerges, research and clinical laboratories may rely on traditional methods, sometimes described as old-fashioned techniques, to promptly respond to the outbreak and describe the biology of the virus. One of these traditional methods is the MNT, the gold standard to answer the important question on the ability to develop protective antibodies against the pathogen.

Consistent with previous reports, in our cohort of symptomatic COVID-19 patients, higher NAb titers were associated with age and disease severity [6,7,8,9,10,11,12,17]. Patients with severe illness (100–200 P/F ratio) had higher peak titers of NAbs than did those with mild illness. Furthermore, patients aged >60 years had significantly higher mean NAbs responses compared to two younger age groups. Thus, it does not appear that older age compromises the development of antibody-mediated immune responses to natural infection. Nevertheless, the role of the other arms of the immune system, including T-cell immunity, has to be taken into account when analyzing the efficacy in the response to SARS-CoV-2 infection. Indeed, uncoordinated humoral and cellular immune response, a factor associated with aging, frequently fails to control disease [7,18,19,20].

Here, we analyzed the kinetics of NAbs in symptomatic COVID-19 patients either hospitalized at INMI, recruited in a follow-up study, or volunteering for CP donation. Different research articles have been published on this topic showing the early development of neutralizing immunity and different kinetics of NAbs in patients followed-up for 20 days to 8 months from symptom onset [13,17,18,21,22,23,24,25,26]. In these studies, NAbs peak during the first month and reach a plateau or, mostly in asymptomatic or paucisymptomatic SARS-CoV-2-infected individuals, decrease down to undetectable levels [13,22,23,24,26]. In a recent publication, the decline observed after the early phase has been associated with the decrease in IgA antibody levels [23]. The role of IgA, IgM, or IgG in the early neutralizing response is nevertheless under investigation, with some studies pointing at IgM and others underlying the role of IgA in this initial phase [25,27,28]. In our study, the analysis of serum samples collected during the first month from symptom onset showed a correlation of neutralization titers with anti-SARS-CoV-2 IgG and IgA, while IgM seemed not to play a role in determining the potency of serum-neutralizing ability.

In our cohort of symptomatic COVID-19 patients, we observed that NAbs are developed as soon as 5 days fso and, after peaking around the first month fso, tend to reach a plateau. Most importantly, NAbs can be detected up to 10 to 11 months fso.

In our longitudinal analysis, we observed different patterns of persistence, with the majority of patients showing stable NAbs over time, while in only 24% of individuals NAb titers declined, and NAbs never reached negative values.

A longitudinal study recently published on the dynamics of neutralizing antibody response in patients with mild, moderate, and severe COVID-19, followed up to six months from symptom onset, identified five patterns of NAb kinetics. In the majority of individuals experiencing mild to moderate disease, the neutralizing response was either not detected or rapidly/slowly waned to low/negative values. The rapid waning was also associated with young age, while the persistent NAb pathway was observed mostly in patients who suffered more severe disease [17].

Our study has some limitations. First, only patients with symptomatic infections have been included, the majority of whom required hospitalization. Second, our data are representative of a single-center longitudinal study, but the consistency with gender, age, and clinical severity suggests that it can be considered representative of a wider patient population. Third, our study was focused on antibody-mediated responses. Probing mucosal and T-cell immunity is important for understanding the immune response of subjects with different clinical severity.

Concerning the long-term duration of immune response after natural infection with SARS-CoV-2, a more comprehensive study on the persistence of both B- and T-cell-mediated immunity 12 months after infection, and more in-depth characterization of immune response kinetics in paucisymptomatic vs. severe COVID-19 patients, is currently underway in our center. The clinical data collected will also help in understanding the different NAb persistence trends observed in longitudinal samples. All these data will help in the interpretation of natural infection and vaccine-induced immune response.

In our opinion, due to the large-scale vaccination campaign, there is an urgent need to identify high-throughput serological tests able to evaluate immunization efficacy and duration.

To date, a number of assays have been developed and evaluated to replace the traditional live-virus-based neutralization test [3] with the aim of avoiding the requirement for a BSL-3 facility and to speed up the laboratory turnaround time (e.g., anti-RBD Ig immunoassays, ACE2-RBD competitive tests, high-throughput serologic platforms, and multiplex assays) [29,30,31,32,33]. However, as we also observed, these tests show a variable correlation to neutralization assays. Moreover, RBD-binding or ACE2-RBD competitive assays are able to reveal an important portion of NAbs but do not measure the neutralizing activity directed against epitopes outside the RBD, such as the N-terminal domain of the S protein [34,35,36,37]. This may lead to an incorrect estimation of functional antibody-mediated immunity, especially in naturally infected individuals, where a more heterogeneous response is observed, as compared to the vaccinee population.

The MNT, although cumbersome and not suitable for routine testing of large sample sizes, remains the gold standard for the assessment of antibody-mediated neutralizing activity upon SARS-CoV-2 infection, and, to date, even the best performing assay shows suboptimal correlation with NAb titers [3]. However, newly developed serological assay platforms may help to provide a pre-screening opportunity to restrict MNT to those samples with the best probability of harboring satisfactory NAb titers.

Smart testing algorithms may assist in optimizing laboratory workflow to monitor antibody-mediated protection. We developed an algorithm for screening CP, based on the analysis of the correlation between MNT and anti-S IgG titers, and established an IgG titer cutoff able to reduce the number of samples undergoing MNT without significant loss of suitable donations.

## Figures and Tables

**Table 1:** Set up of Convalescent Plasma Testing Algorithm.

Anti-S IgG AU/mL	≥1:160/Total	% ≥1:160	% Donor Lost	Spared NAb Tests
0–400	105/402	26.10	0.00	0
15–400	105/391	26.80	0.00	11
60–400	104/315	33.00	0.95	87
80–400	90/254	35.40	14.31	148
100–400	75/213	35.20	28.60	189
150–400	42/127	33.10	60.00	275

Anti-S IgG < 15 AU/mL = negative; AU = Arbitrary Units.

## Data Availability

The data used and/or analyzed during the current study are available, only for sections non-infringing personal information, from the corresponding author on reasonable request.

## Supplementary Materials

The following are available online at https://www.mdpi.com/article/10.3390/v13040655/s1, Figure S1. Anti-SARS-CoV2 immunoglobulins (IgG, IgA, and IgM) correlation to NAb titres during the early phase of infection.

## INMI COVID-19 Laboratory Team

Isabella Abbate, Chiara Agrati, Loredana Aleo, Tonino Alonzi, Alessandra Amendola, Claudia Apollonio, Nicolina Arduini, Barbara Bartolini, Giulia Berno, Silvia Biancone, Angela Bibbò, Licia Bordi, Carla Brega, Marco Canali, Angela Cannas, Maria Rosaria Capobianchi, Fabrizio Carletti, Stefania Carrara, Rita Casetti, Concetta Castilletti, Roberta Chiappini, Lucia Ciafrone, Eleonora Cimini, Sabrina Coen, Francesca Colavita, Rossella Condello, Antonio Coppola, Silvia D’Arezzo, Antonino Di Caro, Stefania Di Filippo, Chiara De Giuli, Lavinia Fabeni, Luisa Felici, Valeria Ferraioli, Federica Forbici, Anna Rosa Garbuglia, Emanuela Giombini, Cesare Ernesto Maria Gruber, Daniele Khouri, Eleonora Lalle, Daniele Lapa, Barbara Leone, Giulia Matusali, Antonio Mazzarelli, Silvia Meschi, Francesco Messina, Claudia Minosse, Claudia Montaldo, Stefania Neri, Carla Nisii, Elisabetta Petrivelli, Fabrizio Petroni, Elisa Petruccioli, Maria Pisciotta, Daniele Pizzi, Gianluca Prota, Gabriella Rozera, Martina Rueca, Rosella Sabatini, Silvia Sarti, Giuseppe Sberna, Roberta Sciamanna, Marina Selleri, Carla Selvaggi, Chiara Stellitano, Antonietta Toffoletti, Silvia Truffa, Federica Turchi, Maria Beatrice Valli, Carolina Venditti, Donatella Vincenti, Antonella Vulcano, Emma Zambelli, Nazario Bevilacqua, Veronica Bordoni, Angela Corpolongo, Alessandra D’Abramo, Giuseppe Ippolito, Luciana Lepore, Andrea Mariano, Emanuele Nicastri, Claudia Palazzolo, Patrizia Lorenzini, Veronica Bordoni, Stefania Notari, Alessandra Sacchi, Laura Scorzolini, Aurora Bettini, Massimo Francalancia, Eliana Specchiarello, Marchetti Federica, D’Alessandro Gaetano, Federici Luigi, Giri Barbara, Iazzetti Roberto, Marafini Giovanni, Mucciante Mirco, Scionti Rachele.
