# Supplementary figures and images for: SARS-CoV-2 Serum Neutralization Assay: A Traditional Tool for a Brand-New Virus
Source: Viruses. 2021 Apr 10;13(4):655. doi: 10.3390/v13040655 (PMC8069482; doi:10.3390/v13040655)

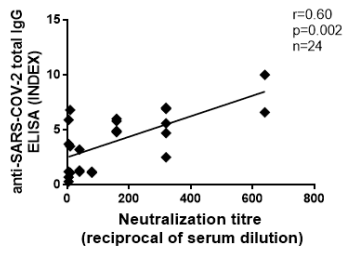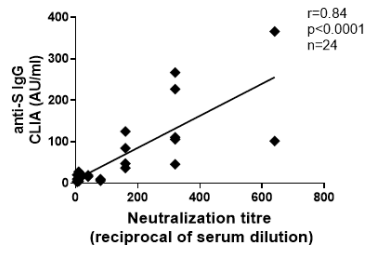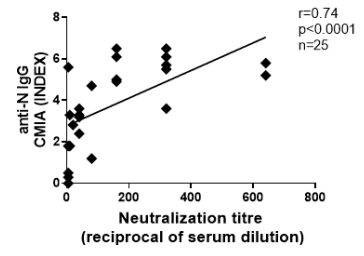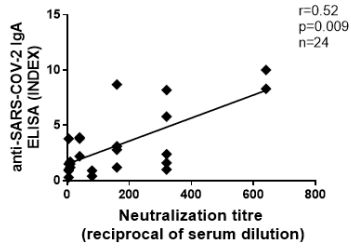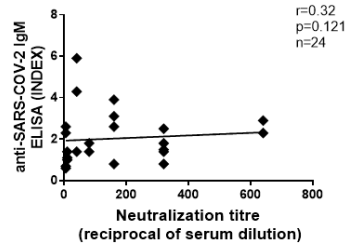

Supplement: Supplementary file 1 [file viruses-13-00655-s001.pdf]
